# Tissue Metabolic Changes Drive Cytokine Responses to *Mycobacterium tuberculosis*

**DOI:** 10.1093/infdis/jiy173

**Published:** 2018-04-03

**Authors:** Ekta Lachmandas, Ana B Rios-Miguel, Valerie A C M Koeken, Eva van der Pasch, Vinod Kumar, Vasiliki Matzaraki, Yang Li, Marije Oosting, Leo A B Joosten, Richard A Notebaart, Mahdad Noursadeghi, Mihai G Netea, Reinout van Crevel, Gabriele Pollara

**Affiliations:** 1 Department of Internal Medicine and Radboud Center for Infectious Diseases, Radboud University Medical Center, Nijmegen; 2 University of Groningen, University Medical Center Groningen, The Netherlands; 3 Laboratory of Food Microbiology, Wageningen University and Research, The Netherlands; 4 Division of Infection and Immunity, University College London, United Kingdom

**Keywords:** tuberculosis, immune response, immunometabolism, metabolism, cytokines, transcriptomics, microarrays, functional genomics, human challenge model

## Abstract

Cellular metabolism can influence host immune responses to *Mycobacterium tuberculosis*. Using a systems biology approach, differential expression of 292 metabolic genes involved in glycolysis, glutathione, pyrimidine, and inositol phosphate pathways was evident at the site of a human tuberculin skin test challenge in patients with active tuberculosis infection. For 28 metabolic genes, we identified single nucleotide polymorphisms that were *trans*-acting for in vitro cytokine responses to *M. tuberculosis* stimulation, including glutathione and pyrimidine metabolism genes that alter production of Th1 and Th17 cytokines. Our findings identify novel therapeutic targets in host metabolism that may shape protective immunity to tuberculosis.

## Teaser

Our study reveals that human tissue challenged with mycobacterial antigens induces differential expression of genes that regulate multiple metabolic pathways. We demonstrate that variable expression of these metabolic genes influences the cytokine response of immune cells stimulated by Mycobacterium tuberculosis.

The activity of many cellular metabolic pathways can impact the host immune response to infections [1]. Individual metabolic pathways have been implicated in antimycobacterial responses: glutathione enhances interleukin-12 (IL-12) and interferon-gamma (IFN-γ) secretion following *Mycobacterium tuberculosis* stimulation [2]; tryptophan catabolism is involved in *M. tuberculosis*-induced production of IL-1β and IL-23 [3] and control of *M. tuberculosis* growth [4]; and a shift towards aerobic glycolysis in *M. tuberculosis*-infected macrophages regulates IL-1β production [5, 6]. However, as metabolic reactions are intrinsically interdependent, the challenge lies in determining the relative roles of these and other pathways during in vivo *M. tuberculosis* infection.

Our group has made use of the human tuberculin skin test (TST) challenge model to faithfully reflect the inflammatory changes that occur at the site of tuberculosis disease, characterizing the tissue immunological pathways induced early after mycobacterial antigen exposure [7]. However, to date, no studies have explored the metabolic changes and their functional consequences on downstream cytokine responses in such a model. Quantitative cytokine production in response to mycobacterial stimulation has been associated with genetic polymorphisms [8]. These cytokine quantitative trait loci (cQTLs) provide a functional insight into how genetics influences an inflammatory response, and in turn identify critical pathways that may be amenable to host-directed therapy. In this study, we use the TST model to test the hypothesis that differential tissue expression of genes involved in regulating metabolic pathways can directly influence cytokine production following *M. tuberculosis* stimulation. In turn, our findings provide putative mechanistic links between the activity of cellular metabolic pathways and immune effector functions.

## METHODS

### Transcriptomic Data Analysis

Transcriptomes were derived from TST and blood of patients with active tuberculosis, and human *M. tuberculosis*-infected and healthy lymph nodes from a separate cohort (Supplementary Table S1). All patients were human immunodeficiency virus (HIV) seronegative and none had diabetes mellitus.

We used the KEGG pathway database (http://www.genome.jp/kegg/pathway.html) to derive 33 pathways associated with human metabolism, yielding a list of 1422 metabolic genes containing no duplicate genes and no annotation to the original pathways (Supplementary Table S2 and Table S3). Bioinformatic analyses were performed as previously described [7] (see Supplementary Methods).

### SNP Extraction and cQTL Mapping

Single nucleotide polymorphisms (SNPs) within metabolic genes and cQTLs were identified as previously described [8] (see Supplementary Methods).

### Metabolite Reporter Analysis

Reporter metabolite analysis [9] was performed in Matlab using the RAVEN Toolbox (http://biomet-toolbox.chalmers.se/index.php?page=downtools-raven) and the human genome-scale metabolic reconstruction network HMR 2.00 provided in Human Protein Atlas (http://www.metabolicatlas.org/downloads/hmr) (see Supplementary Methods).

### Metabolite Depletion Experiments

Peripheral blood mononuclear cells (PBMC) were isolated from 9 healthy Dutch adult volunteers and stimulated with *M. tuberculosis* lysate in the presence or absence of pharmacological manipulators of the glutathione pathway (see Supplementary Methods).

## RESULTS

**Figure 1.**
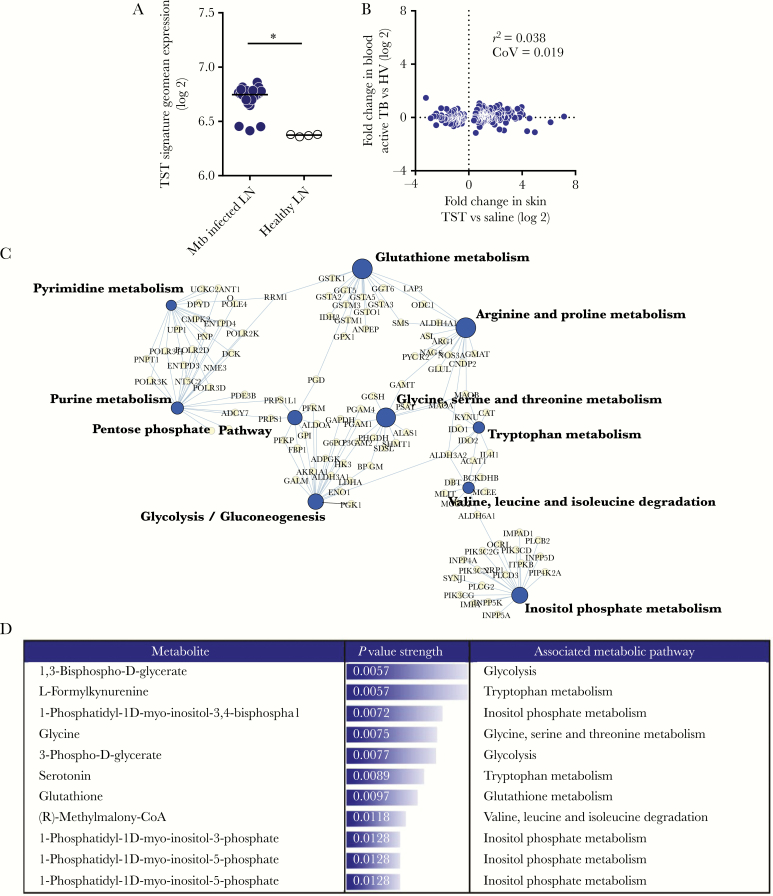
Metabolic pathways enriched in tuberculin skin test (TST) responses relative to saline injection. *A*, Expression of TST signature in *Mycobacterium tuberculosis* (Mtb)-infected lymph nodes (LN) relative to healthy LN. Each dot represents 1 sample. Horizontal lines represent median value expression. * *P* < .0001 by Mann-Whitney test. *B*, Pairwise comparison of 292 genes differentially expressed in TST relative to saline injection compared to the expression difference between the blood of patients with active tuberculosis (TB) and healthy volunteers (HV). *C*, The top 10 most statistically enriched KEGG metabolic pathways in the TST relative to saline injection are represented in a network plot, in which the edges indicate associations between genes (light gray nodes) and named pathways (dark gray nodes), and the node size is proportional to the respective pathway −log10 *P* value enrichment statistic. *D*, Reporter metabolites predicted to be differentially expressed in TST compared to saline injection. Metabolites selected for known association with metabolic pathways and ranked by increasing *P* value. Abbreviations: *r*^2^, Spearman rank correlation coefficient; CoV, covariance.

We have previously demonstrated that the transcriptional response at the site of TST is characterized by upregulation of 1725 genes that closely reflect changes seen in dissected human tuberculosis granuloma relative to healthy lung tissue [7]. We now show that the TST signature is also enriched within *M. tuberculosis*-infected relative to healthy lymph nodes (Figure 1A), confirming that the TST transcriptional responses mirror pathology seen at established sites of human tuberculosis disease.

Out of 1422 metabolic genes, 292 were differentially expressed at the site of TST versus saline injection in patients with active tuberculosis disease (Supplementary Figure S1 and Supplementary Table S4). Expression of these genes at tissue level showed a remarkable lack of concordance with the blood transcriptome of the same patients, as only 9 metabolic genes were differentially expressed in the blood (Figure 1B, Supplementary Figure S2, and Supplementary Table S5). This demonstrates that the TST challenge model provides greater molecular resolution to identify host differential metabolic gene expression to mycobacterial infection than the blood compartment [7].

Given the interconnected relationship between multiple metabolic pathways, we generated a network depicting the 10 most enriched KEGG annotated metabolic pathways based on the 292 differentially expressed metabolic genes (Figure 1C). As expected, the TST induced changes in multiple interlinked metabolic pathways. These included not only previously described pathways such as glycolysis and glutathione metabolism, but also others such as inositol phosphate metabolism and specific amino acid metabolic pathways not previously associated with tuberculosis [1, 2]. Because differentially expressed genes within the same metabolic pathway were both up- or downregulated (Supplementary Figure S3), we evaluated which metabolites in these pathways were most affected using reporter metabolite analysis. This identified metabolites in the human metabolic network around which the most transcriptional changes occur [10]. The model predicted changes in several key metabolites between TST and saline, including 1,3-bisphospho-d-glycerate (glycolysis), l-formylkynurenine (tryptophan metabolism), 1-phosphatidyl-1d-myo-inositol-3,4-bisphosphate, and glutathione (Figure 1D). Therefore, the model predicts that gene expression changes in the TST alter the concentration of several bioactive metabolites in multiple pathways, and that this has the potential to impact the nature of the host immune response to *M. tuberculosis*.

Next, we sought to test the hypothesis that metabolic changes in the tissue environment influence *M. tuberculosis*-induced cytokine production. We used natural genetic variation to identify putative *cis*-acting SNPs for metabolic gene expression and tested whether they might be *trans*-acting SNPs for cytokine responses. We identified metabolic genes differentially expressed in the TST and assessed their impact on cytokine secretion following *M. tuberculosis* lysate stimulation in a cohort of 500 healthy individuals (500FG cohort) [8]. First, we used genotypes extracted from the 1000 Genomes Project to identify 16061 SNPs from the 109 metabolic genes that comprised the 10 most enriched metabolic pathways in the TST (Figure 1C). We then assessed which of these SNPs were associated with variable cytokine secretion, generating 2376 putative cQTLs. To reduce multiple testing false positives, we focused on SNPs in the mRNA coding region of the gene of interest that influence the same gene’s transcription (ie, metabolic gene SNPs that were *cis*-expression QTLs [eQTLs]).

**Figure 2.**
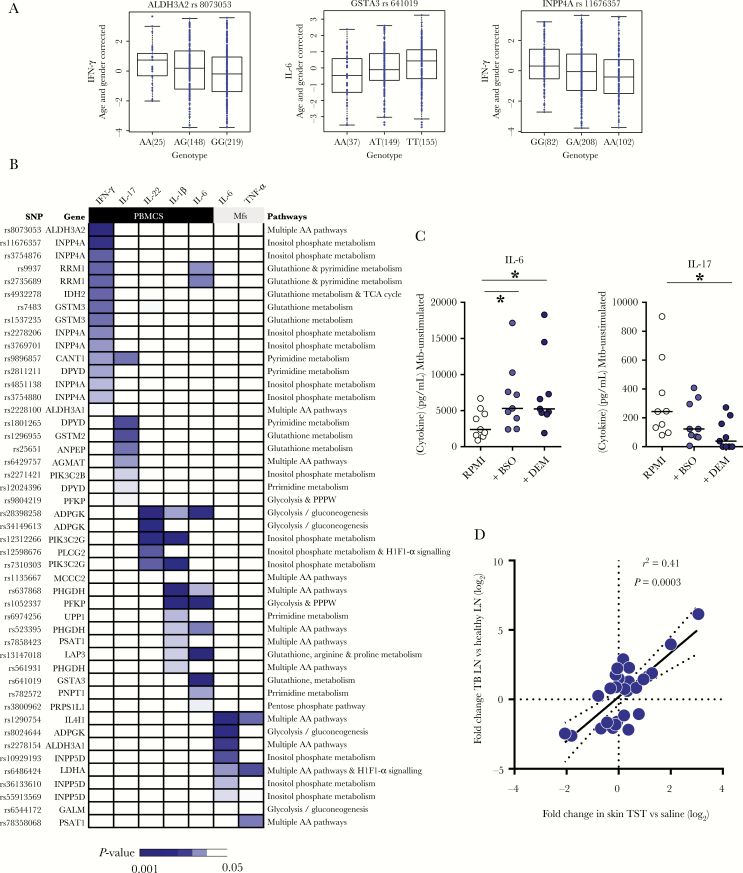
Identification of cytokine quantitative trait loci (cQTLs) within metabolic genes differentially expressed in a tuberculin skin test (TST). *A*, Representative box plots of association between single nucleotide polymorphisms (SNP) genotypes and *Mycobacterium tuberculosis* (Mtb)-induced cytokine levels. Length of the box is the interquartile range and whiskers indicate the range of 1.5 × the length of the box from either end of the box. *P* values were obtained using linear regression analysis of cytokine on genotype data. *B*, Heatmap of all 47 cQTL SNPs and their relationship to cytokine secretion following peripheral blood mononuclear cell (PBMC) or macrophage (Mfs) stimulation with *M. tuberculosis* lysate. *C*, Interleukin-6 (IL-6) and IL-17 secretion from *M. tuberculosis* lysate-stimulated PBMC in the presence or absence of buthionine sulfoximine (BSO) or diethylmaleate (DEM). Horizontal lines represent median value expression. * *P* < .01 Wilcoxon signed-rank test. *D*, Gene expression of 28 metabolic genes with cQTL SNPs in tuberculin skin test (TST) relative to saline injection compared to the expression in *M. tuberculosis*-infected lymph nodes (TBLN) relative to healthy lymph nodes (LN). Abbreviations: IFN-γ, interferon-gamma; *r*^2^, Spearman rank correlation coefficient; RPMI, RPMI 1640 medium; TNF-α, tumor necrosis factor-alpha.

This analysis generated 47 cQTL SNPs from 28 metabolic genes (Figure 2A, 2B, Supplementary Table S6, and Table S7). Based on these genes, the most over-represented metabolic pathways included the glutathione, glycolysis, inositol phosphate metabolism, and pyrimidine pathways (Supplementary Figure S4). Many amino acid metabolism pathways were also observed, and *ALDH3A2*, *ALDH3A1*, *LDHA*, and *IL4I1* were the most frequent constituent genes from these pathways (Supplementary Figure S4). Glutathione and pyrimidine metabolism predominantly influenced the secretion of IFN-γ and IL-17. Seven of 15 (47%) cQTLs that regulated IFN-γ secretion and 4 of 7 (57%) cQTLs that regulated IL-17 secretion were derived from genes assigned to glutathione or pyrimidine metabolic pathways, whereas no cQTLs from these pathways influenced cytokine secretion by macrophages (Figure 2B). In contrast, genes involved in glycolysis, amino acid, and inositol phosphate metabolism acted as cQTLs more ubiquitously, influencing the secretion of both T-cell and myeloid cell-derived cytokines (IL-1β, IL-6, IL-22, and tumor necrosis factor- alpha [TNF-α]) (Figure 2B).

We validated some of the relationships between metabolic changes and cytokine secretion in vitro using buthionine sulfoximine (BSO), an inhibitor of glutathione synthesis, and diethylmaleate (DEM), which depletes cells of glutathione [11]. Both resulted in increased IL-6 and decreased IL-17 secretion by *M. tuberculosis*-stimulated PBMC (Figure 2C), consistent with the cQTL data demonstrating that variable expression of glutathione genes impacted the secretion of both these cytokines (Figure 2B).

Finally, to explore the physiological relevance of the 28 metabolic genes that act as cQTLs, we showed that their expression in *M. tuberculosis*-infected lymph nodes strongly correlated with expression in the TST (Figure 2D), indicating that these metabolic genes have the potential to exert cQTL activity at the site of human tuberculosis disease, thus shaping the local inflammatory response to *M. tuberculosis*.

## Discussion

Individual metabolic pathways have been shown to affect the host response to *M. tuberculosis* [2, 3, 5], but their relative contribution in a multicellular tissue infection setting has not been investigated. We took a systems approach using the human in vivo TST challenge model, revealing gene expression changes in multiple metabolic pathways that in turn predict enrichment of several bioactive metabolites. Genetic polymorphisms in these differentially expressed metabolic genes, as well as pharmacological inhibition, were found to control *M. tuberculosis*-induced cytokine production. Finally, expression of these same genes at the site of tuberculosis disease closely correlated with that observed in the TST, underlying the functional relevance of our findings to the pathogenesis of human tuberculosis infection.

The inflammatory response to TST challenge reflects the immunopathological changes in human tuberculosis disease [7]. Interestingly, in terms of metabolic gene expression, the TST site also closely mirrors *M. tuberculosis*-infected lymph nodes, unlike the peripheral blood of tuberculosis patients, which showed quantitatively smaller changes. Although this may reflect the TST response occurring after acute antigenic challenge in contrast to steady-state assessment in blood, in these same patients the blood transcriptome shows significant deviation from health [7, 12], illustrating that tissue may be the more appropriate context when studying changes in metabolism.

We observed differential gene expression in the TST of multiple metabolic pathways, including glycolysis, glutathione, pyrimidine, and inositol phosphate metabolism. The relevance of changes in the metabolic environment after *M. tuberculosis* infection was supported by the fact that we identified several SNPs in these genes that both act as *cis*-eQTLs and also impact *M. tuberculosis*-induced cytokine secretion by PBMC and macrophages [8], cells that are enriched at the site of TST [7]. For functional validation, we selected one of the pathways identified through gene expression and cQTL analysis, the glutathione pathway, whereby pharmacological inhibition affected *M. tuberculosis*-induced production of IL-6 and IL-17. Importantly, the 500FG cohort data demonstrated that variable expression of genes involved in glutathione metabolism (*GSTM2*, *ANPEP*, *LAP3*, and *GSTA3*) impacted the secretion of these same cytokines. Furthermore, we have previously demonstrated that inhibiting glycolysis using 2-deoxyglucose impaired IL-22 secretion by *M. tuberculosis*-stimulated PBMC, in turn validating the observation from the 500FG data that SNPs in the glycolysis gene *ADPGK* are cQTLs for IL-22 secretion [6].

Our analyses also identified new roles for pyrimidine metabolism, which has recently been associated with regulating inflammasome activity and cytokine secretion [13]. We demonstrate that pyrimidine and glutathione metabolism genes predominantly impact the secretion of T-cell–derived cytokines IFN-γ and IL-17. As such, therapeutic manipulation of pyrimidine and glutathione pathways may influence T helper (T_H_1) and T_H_17 polarization, and thus the balance between protection and pathology in *M. tuberculosis*-infected tissues [14]. In contrast, genes involved in amino acid and inositol phosphate metabolism impact a wider array of cytokines, including those secreted from myeloid cells, which may in part relate to the central role of the metabolite phosphatidylinositol as the backbone of signal transduction components inositol triphosphate (IP3) and protein kinase B (Akt) [6].

Our study has some limitations. Firstly, we restricted our cQTL analyses to SNPs that were also *cis*-eQTLs to limit multiple testing errors, thus likely missing other functionally relevant cQTLs that may act in *trans* via other genes. Equally, identification of SNPs that were eQTLs was limited to databases that probed blood and tissues not infected with *M. tuberculosis*, possibly missing other functionally relevant SNPs in the context of *M. tuberculosis* infection. Furthermore, the effector cytokines studied for cQTL analysis comprise only one facet of the host immune response to *M. tuberculosis* infection and were restricted to the manually selected panel available in the 500FG database, introducing bias into the breadth of immunological effector functions exerted by metabolic gene SNPs. Finally, although reporter metabolite analysis predicted computationally that the gene expression changes impacted on the concentration of several key metabolites, we were not able to assess this directly. The study was conceived retrospectively, and thus tissue samples had already been used wholly for transcriptomic analysis. The relationship between tissue gene expression changes and metabolite concentrations will need to be verified experimentally in future studies.

In summary, this study made a comprehensive assessment of the human tissue metabolic transcriptional response to in vivo mycobacterial antigenic stimulation. A number of known and novel metabolic pathways were differentially expressed, and genetic variation in identified genes affected cytokine responses to *M. tuberculosis*. Therefore, our systems approach provided a novel list of putative metabolic gene cQTLs that are amenable to further experimental validation, and revealed a new layer of complexity to the host antimycobacterial response, supporting the use of host-directed strategies that target cellular metabolism, such as the regulation of glycolysis by metformin [15]. Furthermore, our approach combining transcriptomics and functional genomics illustrates a pipeline that can be used to identify novel and clinically relevant pathways in the context of other infectious diseases.

## Supplementary Data

Supplementary materials are available at *The Journal of Infectious Diseases* online. Consisting of data provided by the authors to benefit the reader, the posted materials are not copyedited and are the sole responsibility of the authors, so questions or comments should be addressed to the corresponding author.

Supplementary Lachmandas Figure S1

Supplementary Lachmandas Figure S2

Supplementary Lachmandas Figure S3

Supplementary Lachmandas Figure S4

Supplementary Lachmandas Table S1

Supplementary Lachmandas Table S2

Supplementary Lachmandas Table S3

Supplementary Lachmandas Table S4

Supplementary Lachmandas Table S5

Supplementary Lachmandas Table S6

Supplementary Lachmandas Table S7

Supplementary Methods

Supplementary Figure Legends

## Notes

***Financial support.*** This work was supported by the Wellcome Trust Research Fellowship to G. P. (grant number WT101766/Z/13/Z); Wellcome Trust Investigator Award to M. N. (grant number 207511/Z/17/Z); the National Institute for Health Research Biomedical Research Centre at University College London Hospital; by a European Research Council Consolidator Grant to M. G. N. (grant number 310372); and a Spinoza grant of the Netherlands Organization for Scientific Research (to M. G.). N. R. v. C. and E. L. were supported by the European Union’s Seventh Framework Programme project TANDEM (grant number HEALTH-F3-2012–305279).

***Potential conflicts of interest.*** All authors: No reported conflicts.. All authors have submitted the ICMJE Form for Disclosure of Potential Conflicts of Interest. Conflicts that the editors consider relevant to the content of the manuscript have been disclosed.
