# Supplementary figures and images for: Tissue Metabolic Changes Drive Cytokine Responses to Mycobacterium tuberculosis
Source: J Infect Dis. 2018 Apr 3;218(1):165–70. doi: 10.1093/infdis/jiy173 (PMC5989606; doi:10.1093/infdis/jiy173)

## Slide 1
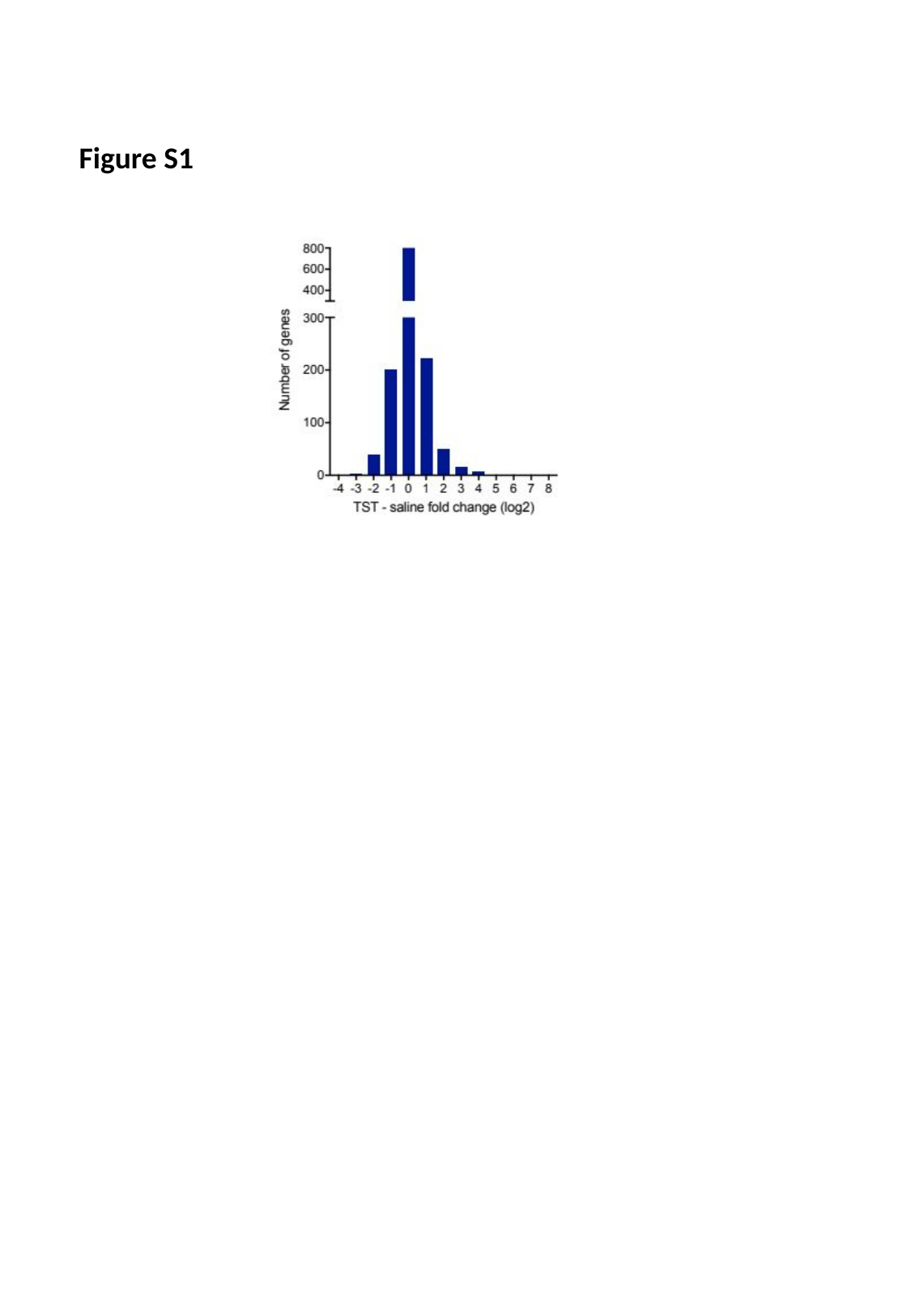

Figure S1

Supplement: Supplementary Lachmandas Figure S1 [file jiy173_suppl_supplementary_lachmandas_figure_s1.pptx]

## Slide 1
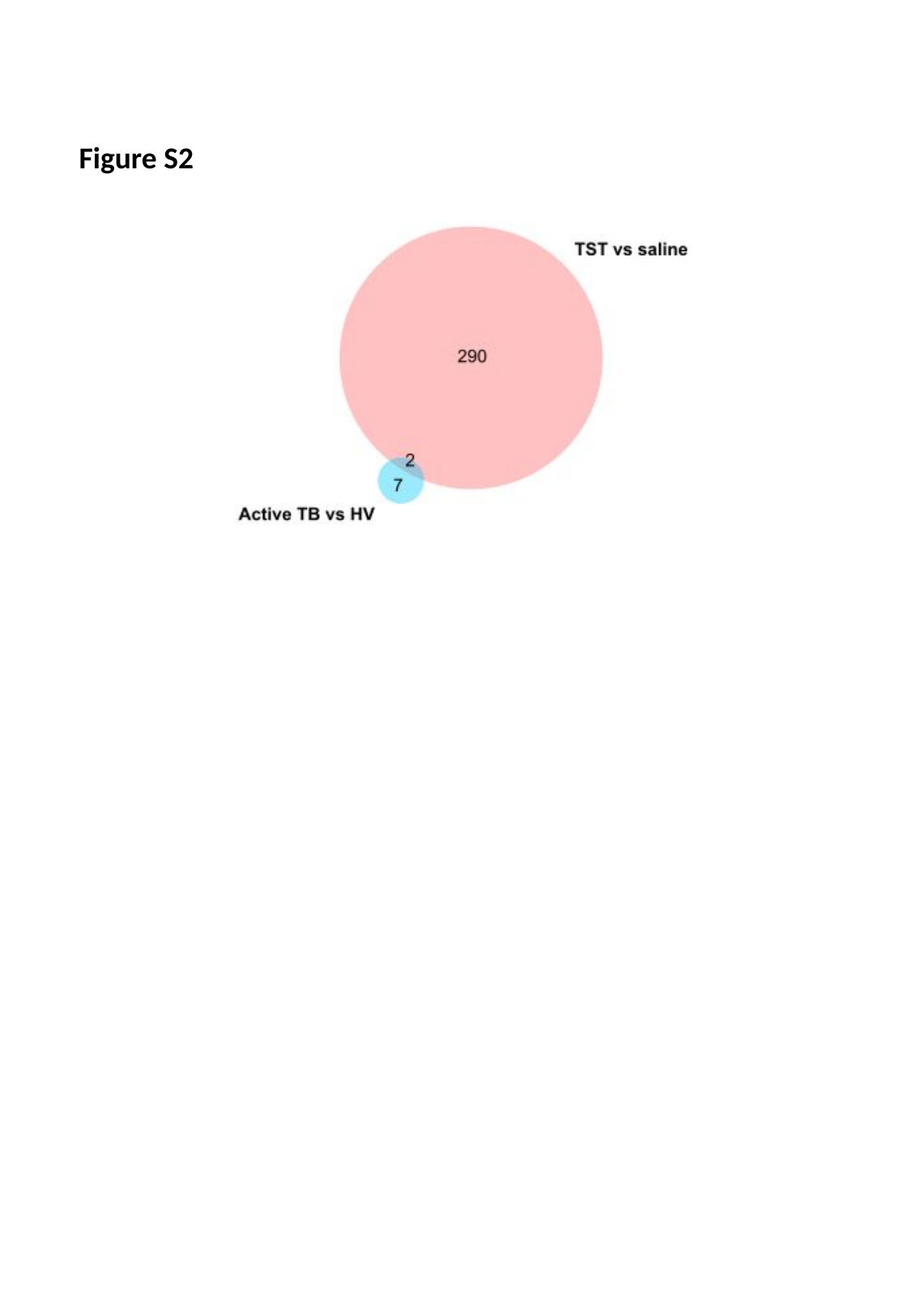

Figure S2

Supplement: Supplementary Lachmandas Figure S2 [file jiy173_suppl_supplementary_lachmandas_figure_s2.pptx]

## Slide 1
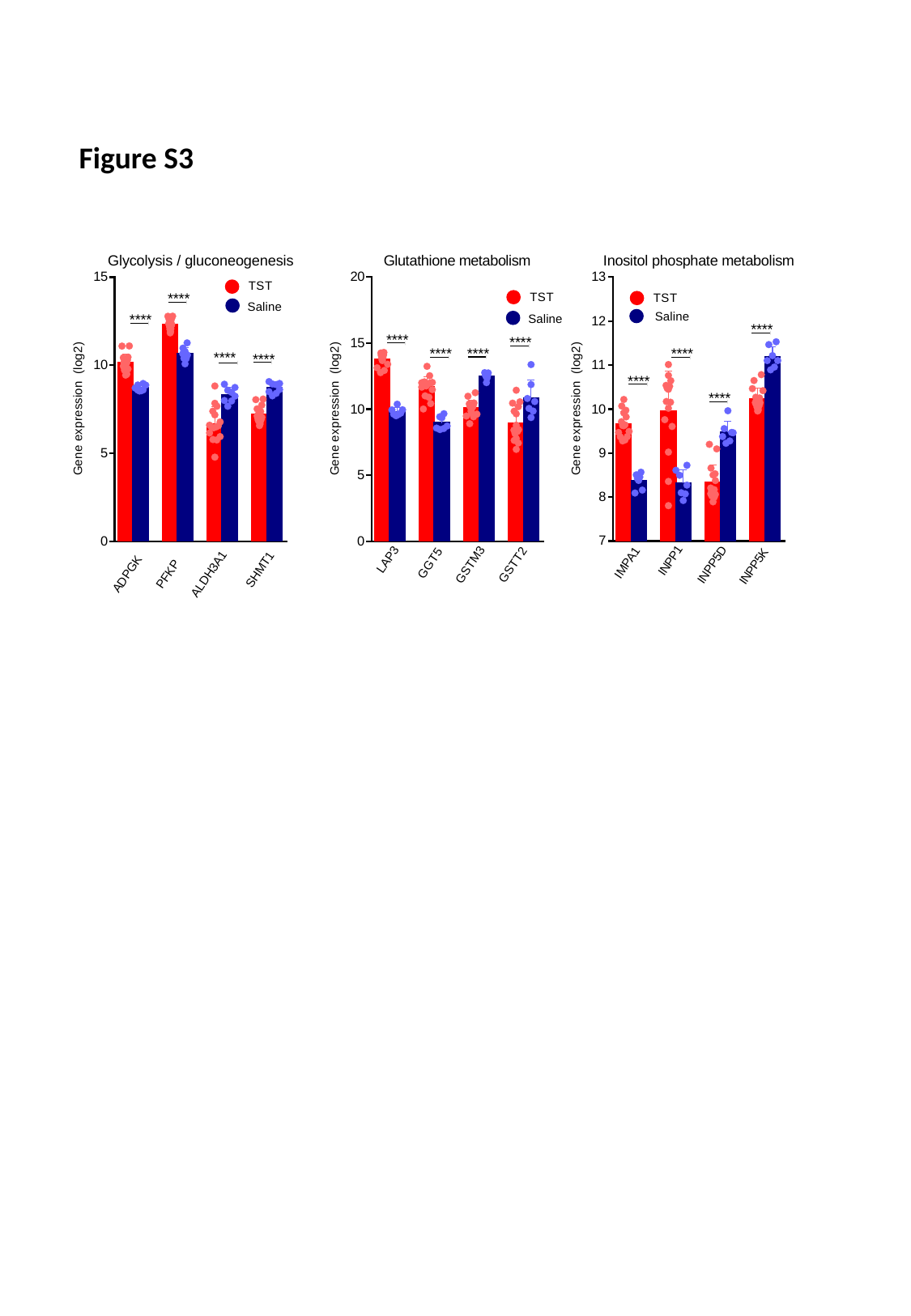

Figure S3

Supplement: Supplementary Lachmandas Figure S3 [file jiy173_suppl_supplementary_lachmandas_figure_s3.pptx]
