# Supplementary material for: Tissue Metabolic Changes Drive Cytokine Responses to Mycobacterium tuberculosis
Source: J Infect Dis. 2018 Apr 3;218(1):165–70. doi: 10.1093/infdis/jiy173 (PMC5989606; doi:10.1093/infdis/jiy173)
Supplement: Supplementary Lachmandas Figure S4 [file jiy173_suppl_supplementary_lachmandas_figure_s4.pptx]

## Slide 1
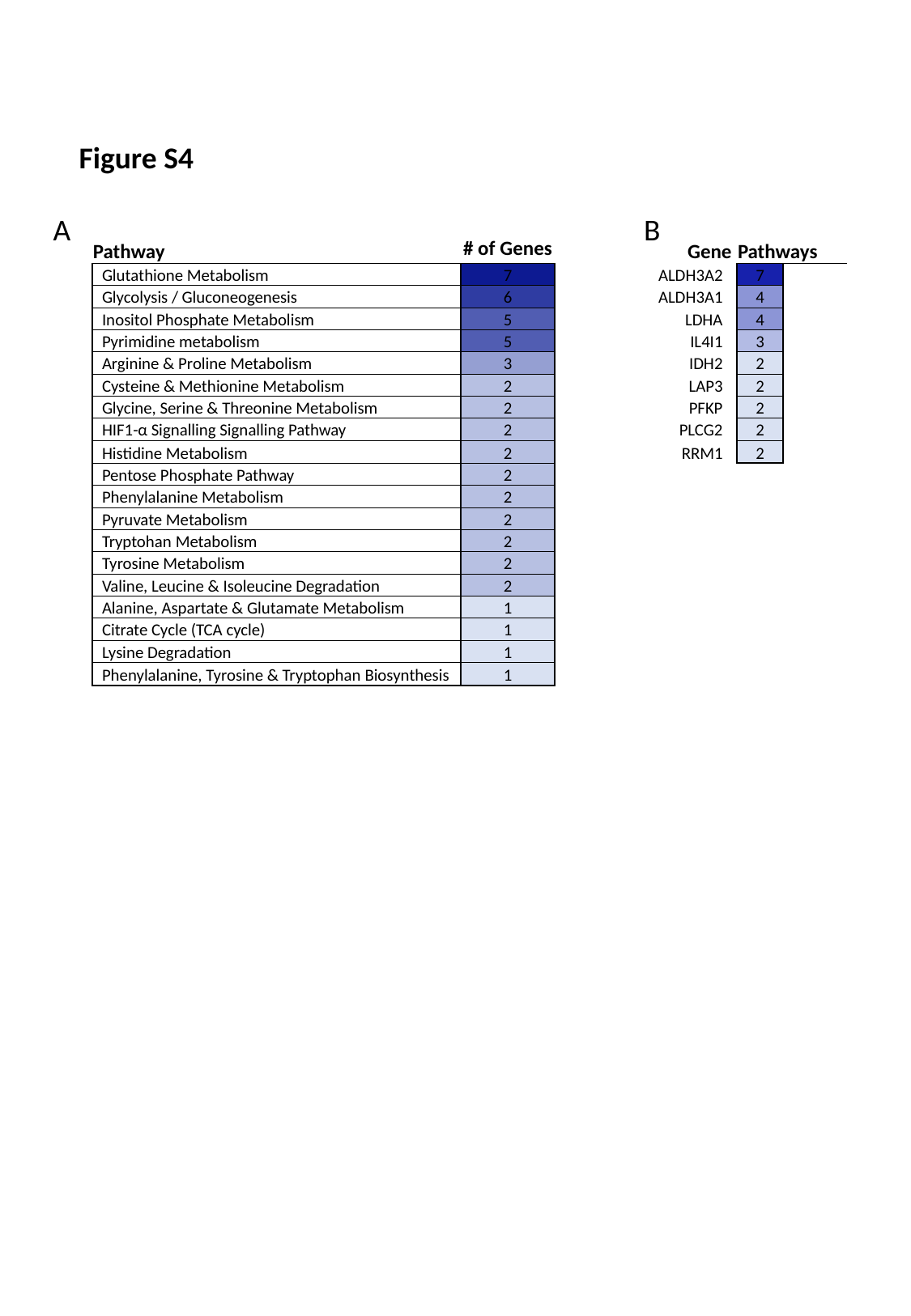

Figure S4
A
B
| Pathway | # of Genes | | Gene | Pathways | |
| --- | --- | --- | --- | --- | --- |
| Glutathione Metabolism | 7 | | ALDH3A2 | 7 | |
| Glycolysis / Gluconeogenesis | 6 | | ALDH3A1 | 4 | |
| Inositol Phosphate Metabolism | 5 | | LDHA | 4 | |
| Pyrimidine metabolism | 5 | | IL4I1 | 3 | |
| Arginine & Proline Metabolism | 3 | | IDH2 | 2 | |
| Cysteine & Methionine Metabolism | 2 | | LAP3 | 2 | |
| Glycine, Serine & Threonine Metabolism | 2 | | PFKP | 2 | |
| HIF1-α Signalling Signalling Pathway | 2 | | PLCG2 | 2 | |
| Histidine Metabolism | 2 | | RRM1 | 2 | |
| Pentose Phosphate Pathway | 2 | | | | |
| Phenylalanine Metabolism | 2 | | | | |
| Pyruvate Metabolism | 2 | | | | |
| Tryptohan Metabolism | 2 | | | | |
| Tyrosine Metabolism | 2 | | | | |
| Valine, Leucine & Isoleucine Degradation | 2 | | | | |
| Alanine, Aspartate & Glutamate Metabolism | 1 | | | | |
| Citrate Cycle (TCA cycle) | 1 | | | | |
| Lysine Degradation | 1 | | | | |
| Phenylalanine, Tyrosine & Tryptophan Biosynthesis | 1 | | | | |
| | | | | | |
