# Supplementary material for: Tissue Metabolic Changes Drive Cytokine Responses to Mycobacterium tuberculosis
Source: J Infect Dis. 2018 Apr 3;218(1):165–70. doi: 10.1093/infdis/jiy173 (PMC5989606; doi:10.1093/infdis/jiy173)
Supplement: Supplementary Methods [file jiy173_suppl_supplementary_methods.docx]

## Supplementary Methods

### Transcriptomic data analysis

Transcriptomic data from TST and blood of patient with active TB were derived from datasets E-MTAB-3254 and E-MTAB-3260 (<https://www.ebi.ac.uk/arrayexpress/>). This comprised 16 individuals with active TB disease who underwent both TST skin biopsy and blood transcriptome profiling. We also included 8 individuals with active TB who received saline skin injection as a comparator for the TST transcriptomic response. Additionally, the blood transcriptome of 8 healthy volunteers was analysed to assess the transcriptomic changes in the blood of the patients with active TB. Transcriptomes from human Mtb-infected (n=22) and healthy lymph nodes (LN) (n=4) were derived from a separate cohort of patients (dataset E-GEOD-63548). In all datasets used, patients were HIV seronegative and none had diabetes mellitus. Links to the publications that first presented these datasets are available in table S1, providing more detailed information on the tissue specimens, storage conditions as well as patient demographics.

For all datasets, probe identifiers were converted to gene symbols using platform annotations provided with each dataset. Datasets were sorted alphabetically by gene symbol, and duplicate genes were removed using Microsoft Excel duplicate remover function. Significant differences in metabolic gene expression between groups was performed by unpaired t-test with alpha p<0.01 and Bonferroni correction for multiple testing in Multi-Experiment Viewer software (<http://www.tm4.org/)>. Pathway enrichment using InnateDB online tool was performed on the resulting gene list (<http://innatedb.com/>). Genes contributing to the 10 most enriched KEGG metabolic pathways were put forward for SNP and QTL analyses. Network plots were generated using Gephi v0.8.2 (<https://gephi.org/>), and Venn diagrams were constructed using BioVenn tool (<http://www.cmbi.ru.nl/cdd/biovenn/>).

### SNP extraction and cQTL mapping

Single nucleotide polymorphisms (SNPs) within metabolic genes were identified from the publicly available variation database dbSNP using the NCBI Variation Viewer tool ([www.ncbi.nlm.nih.gov/variation/view](http://www.ncbi.nlm.nih.gov/variation/view)). SNPs located 250 kb upstream and downstream of the genes of interest with minor allele frequency ≥ 0.05 were extracted using the GRCh38.p7 assembly. Linkage disequilibrium (LD) SNP pruning using genotypes extracted from 1000 Genomes Project for Europeans (<http://www.internationalgenome.org/home>) based on pairwise genotypic correlation was performed using plink v1.07 (<http://zzz.bwh.harvard.edu/plink/dataman.shtml>) and one of a pair of SNPs was removed if the LD was greater than 0.5. LD-pruned SNPs as well as any other functional SNP (missense variants) that were removed due to high LD were then mapped (p<0.05 cut-off) for cQTLs in the 500FG cohort. This cohort consists of 500 healthy individuals of Dutch European ancestry from the Human Functional Genomics Project ([www.humanfunctionalgenomics.org](http://www.humanfunctionalgenomics.org)), from whom peripheral blood mononuclear cells (PBMC) or macrophages have been stimulated for cytokine production with heat-killed Mtb (1μg/ml) and the secretion of IFNγ, IL-17 and IL-22 (7 days post-stimulation), and IL-1β, IL-6 and TNFα (24hr post-stimulation) measured, as previously described [1]. To identify cQTLs, raw cytokine levels were first log2 transformed then mapped to genotype data using a linear regression model with age and gender as covariates. P values were obtained using linear regression analysis of cytokine on genotype data, as previously described [1]. To reduce multiple testing false positives, the putative cQTLs were further refined to include only SNPs that both were present within the coding region of the gene of interest (<https://genome.ucsc.edu/>) and had eQTL activity for the encoded gene (<http://www.gtexportal.org/home/>).

### Metabolite reporter analysis

Reporter metabolite analysis [2] was performed in Matlab using the RAVEN Toolbox (<http://biomet-toolbox.org/index.php?page=downtools-raven>) and the human genome-scale metabolic reconstruction network HMR 2.00 provided in Human Protein Atlas (<http://www.metabolicatlas.org/downloads/hmr>). The algorithm represents the input network as a bipartite undirected graph where both enzymes and metabolites form nodes and the interactions between them represent edges. The model calculates an enrichment score for each metabolite in the graph based on the normalized differential expression data of its neighbouring enzymes seen in Table S3.

### Metabolite depletion experiments

PBMCs isolation from buffy coats from 9 healthy Dutch adults (Sanquin Bloodbank, Nijmegen, the Netherlands) (estimated tuberculosis incidence 1.5/100.000) was performed by differential centrifugation over Ficoll-Paque™ PLUS (GE Healthcare Biosciences). Cells were re-suspended in RPMI^+^ (RPMI 1640 (Gibco) supplemented with 10 𝜇g/mL gentamicin (Lonza), 2 mM GlutaMAX (Life Technologies), and 1 mM pyruvate (Life Technologies) and adjusted to 5 x 10^6^ PBMCs/mL. 100 μL of PBMCs were stimulated with RPMI^+^ or 1 μg/mL *Mycobacterium tuberculosis* strain H37Rv lysate, in the presence or absence of 100 µM DL-buthionine-sulfoximine (BSO) or 50 µM Di-ethyl maleate (DEM) (both from Sigma) for 24 h or together with 10% pooled human serum for 7 days [8]. Cell culture supernatants were collected and stored at −20°C. Cytokines in culture supernatants were measured by commercial ELISA kits for IL-17A and IL-6 (R&D Systems). Lactate dehydrogenase (LDH) levels were also measured in the supernatant of cultured PBMC to assess cellular viability using commercial ELISA kits (Promega). Neither BSO nor DEM resulted in significant increase in LDH release in treated cells compared to untreated cells following 24 hours of culture (data not shown).

## Supplementary methods references

1. Li Y, Oosting M, Smeekens SP, et al. A Functional Genomics Approach to Understand Variation in Cytokine Production in Humans. Cell. **2016**; 167(4):1099–1110.e14.

2. Patil KR, Nielsen J. Uncovering transcriptional regulation of metabolism by using metabolic network topology. Proc Natl Acad Sci U S A. **2005**; 102(8):2685–2689.
