# Supplementary material for: Tissue Metabolic Changes Drive Cytokine Responses to Mycobacterium tuberculosis
Source: J Infect Dis. 2018 Apr 3;218(1):165–70. doi: 10.1093/infdis/jiy173 (PMC5989606; doi:10.1093/infdis/jiy173)
Supplement: Supplementary Figure Legends [file jiy173_suppl_supplementary_figure_legends.docx]

**Supplementary figure legends:**

**Figure S1: Metabolic gene expression in TST.**

Frequency distribution of fold increases and decreases in metabolic gene transcript in TST compared to saline injections.

**Figure S2: Differential metabolic gene expression in tissue and blood.**
Venn diagram identifying the number of metabolic genes differentially expressed between TST and saline skin injection (red) and between the blood of patients with active TB disease and healthy volunteers (HV) (blue).

**Figure S3: Metabolic genes assigned to individual pathways show both increased and decreased enrichment in the TST.**

Bar graphs representing gene expression of select metabolic genes in TST (red) and saline (blue) groups. Data represented as mean ± SEM from patients with active TB who received a TST (n=16) or saline injection (n=8). **** p<0.0001 (Mann-Whitney test)

**Figure S4: Relationship between metabolic pathway and cQTL genes identified.**

(A) Number of cQTL genes comprising each of the represented metabolic pathways. (B) Number of genes representing amino acid metabolism pathways.
